# Conduction abnormalities and ventricular arrhythmogenesis: The roles of sodium channels and gap junctions

**DOI:** 10.1016/j.ijcha.2015.10.003

**Published:** 2015-10-30

**Authors:** Gary Tse, Jie Ming Yeo

**Affiliations:** a School of Biomedical Sciences, Li Ka Shing Faculty of Medicine, University of Hong Kong, Hong Kong; b School of Medicine, Imperial College London, SW7 2AZ, UK

**Keywords:** Conduction, Conduction velocity, Ventricular arrhythmia, Sodium channel, Gap junction

## Abstract

Ventricular arrhythmias arise from disruptions in the normal orderly sequence of electrical activation and recovery of the heart. They can be categorized into disorders affecting predominantly cellular depolarization or repolarization, or those involving action potential (AP) conduction. This article briefly discusses the factors causing conduction abnormalities in the form of unidirectional conduction block and reduced conduction velocity (CV). It then examines the roles that sodium channels and gap junctions play in AP conduction. Finally, it synthesizes experimental results to illustrate molecular mechanisms of how abnormalities in these proteins contribute to such conduction abnormalities and hence ventricular arrhythmogenesis, in acquired pathologies such as acute ischaemia and heart failure, as well as inherited arrhythmic syndromes.

## 1 Introduction

Cardiac arrhythmias arise from disruptions in the normal orderly sequence of electrical activation and recovery of the heart. On the one hand, ventricular arrhythmias can lead to sudden cardiac death (SCD), which accounts for around 400,000 deaths in the United States [1], [2], [3]. This represents a prevalence of 0.1% in the population. On the other hand, atrial arrhythmias are the most common arrhythmias observed in clinical practice [4], affecting around 2 million people in the U.S. [5]. It is a major contributor to cardiovascular morbidity. For example, up to 15% of all strokes in the U.S. can be attributed to atrial fibrillation [6].

Ventricular arrhythmias can be categorized into disorders affecting cellular depolarization or repolarization [7], but can also be caused by abnormalities in action potential conduction [8]. The commonest underlying mechanism is the formation of re-entry circuits [9]. Re-entry occurs when an action potential fails to extinguish itself and re-activates a region that has recovered from refractoriness [10], [11]. It may occur in the presence of an obstacle, around which an action potential can travel (circus-type) [12], or without an obstacle (reflection or phase 2) [13], [14]. For circus re-entry to occur, three criteria must be met. First, there must be an obstacle around which an action potential (AP) can circulate. This need not to be a structural abnormality, but can be a functional core of refractory tissue. Secondly, conduction velocity (CV) must be sufficiently reduced so that the myocardial tissue ahead of the excitation wavefront remains excitable. Finally, unidirectional conduction block must be present to prevent waves from self-extinguishing. These three conditions form part of the arrhythmogenic substrate, which upon a trigger, for example from premature generation of an AP, can serve to sustain re-entry. Recent review articles have focused on the mechanisms of cardiac conduction [15] and the factors governing myocardial CV [8], [16]. This article briefly discusses the factors causing conduction abnormalities, as well as the roles that sodium channels and gap junctions play in AP conduction and how abnormalities in these proteins contribute to ventricular arrhythmogenesis.

## 2 Reduced conduction velocity and unidirectional conduction block

**Fig. 1:**
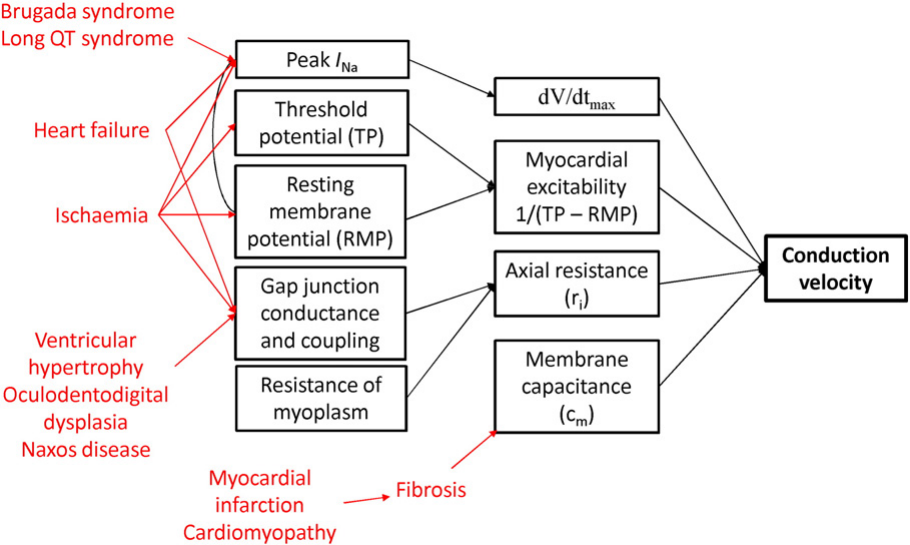
Determinants of conduction velocity, and the effects of different pathological conditions on these.

CV of the propagating APs depends on both active and passive properties of the cell membrane (Fig. 1). Active properties refers to voltage-dependent conductances, mainly mediated by *I*_Na_, which determines the AP upstroke (phase 0) [18]. A faster CV can arise from two situations. Firstly, a higher maximum upstroke velocity, dV/dt_max_ brings the membrane to threshold much more quickly. Secondly, increased myocardial excitability given by 1/(threshold potential–resting membrane potential) means that smaller inward currents are required to reach threshold. The resting membrane potential also influences *I*_Na_ through regulation of the inactivation kinetics of sodium channels [19], [20], [21], [22], [23]. In contrast, passive properties depend on capacitive and resistive components of the cell membrane as well as the myocardial architecture [24]. Axial resistance (r_i_) depends on the resistance of both the myoplasm [25] and the gap junctions between myocytes [26]. Decreased r_i_ would increase CV. Membrane capacitive load is determined by the surface area and volume ratio [24]. Increased membrane capacitance (C_m_) prolongs the time needed to bring membrane to threshold, thereby reducing CV. These processes determining CV can be described mathematically by the cable theory [27]. However, recent experimental data have challenged the applicability of this theory to cardiac conduction [28], and have attracted ongoing debate [29], [30]. Traditional cable theory does not explicitly consider junctional clefts in the extracellular space and is therefore incompatible with the notion of ephaptic coupling: interested readers should be directed to this article here [31]. As shown in Fig. 1, acquired and congenital heart diseases influence various determinants of CV, which will be discussed in turn.

Additional factors can modulate the CV of the AP wave. Firstly, gap junctions, high-resistance ion channels located between successive myocytes [32], [33] cause conduction slowing and discontinuous propagation [34], [35]. Secondly, the complex tissue architecture of the myocardium leads to anisotropic conduction, with greater velocity in the longitudinal compared to the transverse direction [36], [37]. This is in part due to higher density of gap junctions at the ends of myocytes than in the lateral margins [38]. Finally, the source and sink are determined by the plasma membrane excitability and tissue-structure properties, respectively [8]. Interactions between these factors are recognized to cause reduced CV, conduction block and fractionation of the excitation wave [39]. Finally, it is also important to appreciate that conduction and repolarization are not independent processes, but one can affect the other. For example, *I*_to_ inhibition enhances conduction between cell pairs, suggesting that repolarization currents are able to influence CV [40], [41]. The accepted view that voltage-gated sodium channels, gap junctions and desmosomes are separate entities with distinct functions has been challenged. Sodium channels were assumed to be separate from gap junctions but were later found in intercalated disks [42], co-localizing with gap junctions and playing an important role in cardiac conduction [43]. This co-localization may constitute a cardiac ephapse, thereby contributing to ephaptic coupling [44]. Its discussion is beyond the scope of the current paper and interested readers are referred to review articles here [45], [46].

Unidirectional conduction block can occur in homogeneous cardiac tissues in the presence of functional asymmetries [8]. Firstly, it can arise from interaction between the activation wavefront and the refractory tail [47]. This can occur with premature initiation of an AP, e.g. from early or delayed afterdepolarization phenomena, or co-existence of interacting wavefronts during multiple wavelet re-entry [8]. Secondly, heterogeneities in membrane excitability increase the vulnerability to unidirectional block. This could occur in regional ischaemia in which there is local accumulation of potassium ions [48], causing both a positive shift in the resting membrane potential and inhomogeneities in sodium channel recovery [49]. Thirdly, increased spatial heterogeneities in refractoriness can arise from alterations in depolarizing or repolarizing currents; for example, this can occur with regional ischaemia where extracellular accumulation of potassium produces shortening of the AP [49].

## 3 Sodium channels

Voltage-gated sodium channels are responsible for the initiation and propagation of APs in excitable cells. Each channel consists of a pore-forming α-subunit, a modulatory β-subunit and other regulatory proteins. Of these, the Na_V_1.5 α-subunit is encoded by the *SCN5A* gene [50] and is made of four domains (I to IV) with each domain containing six transmembrane segments (S1 to S6). Upon depolarization, the S4 segments, which are positively charged, undergo outward movement [51], [52]. This opens the channel pore and allows the influx of sodium ions. The resulting transmembrane current, *I*_Na_, therefore determines myocardial excitability and CV of the APs. Depolarization also initiates fast inactivation [53], [54]. The mechanism involves the linker region between domains III and IV, which functions as a ‘lid’ to occlude the pore [55], [56], [57], [58]. Slow inactivation involves the P-segment linker sequence between S5 and S6 bending back into the membrane and lining the outer pore [59], [60]. Binding sites for Ca^2 +^ and the Ca^2 +^-binding protein calmodulin are present at the carboxyl terminus [65], [66], allowing modulation of channel function [67], [68]. Ca^2 +^/calmodulin-dependent Kinase II phosphorylates the sodium channel, causing a negative shift in the voltage-dependence of steady-state inactivation without altering the voltage-dependence of steady-state activation or the peak *I*_Na_ [69].

*I*_Na_ consists mainly of a tetrodotoxin-insensitive component, attributable to the cardiac isoform Na_V_1.5 [50], [70], [71]. It also contains a persistent, tetrodotoxin-sensitive component, as suggested initially by voltage clamp technique [61], [62] and later confirmed by patch clamping [63], [64]. This is mediated by neuronal isoforms Na_V_1.1, Na_V_1.3, and Na_V_1.6 [72], [73], [74]. Recent immunohistochemical experiments have demonstrated neuronal isoforms in cardiac tissue of many species, in keeping with the electrophysiological findings [75]. They were found in pacemaker cells with whole cell current and voltage clamp experiments demonstrating a contributory role in pacemaker activity [76]. Furthermore, they were localized to the transverse tubular system of cardiomyocytes, and tetrodotoxin application resulted in desynchronization of excitation-contraction coupling and a decrease in cardiac contractility [77]. However, this is a contentious area as another study showed no significant effects of tetrodotoxin on shortening of rat ventricular myocytes [78].

### 3.1 Abnormalities in sodium channel structure and function

Two main types of autosomal dominant inherited arrhythmias resulting from mutations in *SCN5A* have been described [79]. Firstly, gain-of-function mutations are found in Long QT Syndrome (LQTS) type 3 [80]. These produce disruptions in fast inactivation and allow repeated channel opening during sustained depolarization [81], [82], [83]. Normally, the repolarization time course is determined by the balance between the inward currents mediated by the voltage-gated L-type calcium channel *(I*_Ca,L_) and sodium–calcium exchanger (*I*_NCX_), and the outward currents mediated by the voltage-gated delayed rectifier potassium channels (*I*_K_) [84]. The rapid and slow currents (*I*_Kr_ and *I*_Ks_, respectively) make up *I*_K_. Persistent sodium current activation leads to delayed cellular repolarization and the development of early after-depolarization phenomena by the two mechanisms. Firstly, depolarizing shifts in the membrane potential can reactivate the L-type calcium channels [85], resulting in increased *I*_Ca__,L_ that further depolarizes the membrane. This sets up a positive feedback loop, triggering an AP. Secondly, at membrane potentials negative to the threshold of *I*_Ca__,L_ activation (but before full repolarization), spontaneous calcium release from the sarcoplasmic reticulum can activate *I*_NCX_ [86], resulting in membrane depolarization. Aberrant initiation of APs themselves could produce all three conditions for re-entry: unidirectional block, slowed conduction and a refractory core around which an AP can circulate [87]. Interestingly, in sudden unexplained death in epilepsy (SUDEP), a possible explanation is ventricular arrhythmias [88], [89]. The proposed mechanism is AP prolongation due to increased contributions of neuronal sodium channel isoforms to the late component of *I*_Na_ (*I*_Na, L_), similar to that of LQTS type 3 [88], [89].

Secondly, loss-of-function mutations in the *SCN5A* gene are observed in Brugada syndrome [90]. These have opposing effects on the fast and slow inactivation of sodium channels [91]. Fast inactivation is disrupted, culminating in a sustained sodium current, which prolongs repolarization at slow heart rates. However, the intermediate kinetic component of slow inactivation is augmented, delaying sodium channel recovery and reducing the sodium current at fast heart rates. Normally, *I*_Na_ is balanced by the transient outward potassium current, *I*_to_ in the early plateau phase of the AP. Because higher *I*_to_ density is observed in the epicardium compared to the endocardium, it has been postulated that repolarization occurs more readily in the epicardium, resulting in a loss of dome morphology [92]. Propagation of the AP dome from regions where it is maintained to regions where it is abolished can produce an extrasystole that initiates ventricular tachycardia (VT) [93], [94]. Although traditionally linked to abnormal repolarization, slowed AP and discontinuous conduction have recently been recognized as contributors towards arrhythmogenesis in Brugada syndrome [93], [95].

In contrast, autosomal recessive mutations of *SCN5A* have been associated with the development of Sick Sinus Syndrome (SSS) [96]. Progressive cardiac conduction defect (PCCD), also called Lenègre disease, has been linked to mutations of *SCN5A* [97], [98], [99]. This is characterized by progressive alterations in conduction with bundle branch blocks potentially leading to complete atrio-ventricular block. Scn5a^+/−^ mice modelling Lenègre disease showed progressive conduction defects associated with myocardial fibrosis [100]. Finally, overlap disorders have been described in several *SCN5A* mutations [101]. Most recently, a case involving p.Y1449C mutation was reported with phenotypes of conduction disease, Brugada syndrome and atrial flutter [102]. Other phenotypes have also been described, which can be explained by the biophysical effects produced by the particular mutation in the *SCN5A* gene [103].

In addition to the above congenital abnormalities in *SCN5A*, acquired alterations in channel properties have been observed. Experimental models of heart failure have demonstrated positive shifts in the resting membrane potential, which causes partial inactivation of sodium channels [104]. Furthermore, deglycosylation of the sodium channels has been detected, in turn causing a positive shift in the voltage-dependence of steady-state activation and a negative shift in the voltage dependence of steady-state inactivation [105]. The net effect is a reduced transient component of the sodium current (*I*_Na, T_), peak *I*_Na_ leading to a decrease in dV/dt_max_. Increased late component of *I*_Na_ (*I*_Na, L_) has been observed in heart failure and also in post-infarction modelling, leading to action potential prolongation [106], [107], [108], [109]. In the failing myocardium, calcium handling is abnormal [110], with reduced amplitudes of Ca^2 +^ transient, sarcoplasmic reticular Ca^2 +^ content and Ca^2 +^ removal [111]. Calmodulin Kinase II is upregulated and its activity increases during heart failure [112], which would be expected to increase *I*_Na, L_ [110]. This persistent activation of *I*_Na_ is likely to be a contributing factor for sudden death in patients with failing ventricles.

A particular type of potassium channel, K_ATP_, is regulated by ATP levels inside the cells [113]. Under normoxic conditions, they are strongly inhibited by intracellular ATP [114]. In acute ischaemia, the oxygen supply cannot match the metabolic demand of the myocardial tissue, resulting in depletion of ATP and switch to anaerobic pathway for substrate utilization. K_ATP_ channels are activated upon ATP depletion and ADP accumulation [114], producing action potential shortening [115]. However, other experiments have demonstrated no action potential shortening during ischaemia, suggesting that K_ATP_ channels might not have opened at least during early ischaemia [116]. Furthermore, a number of extracellular and intracellular changes are observed during myocardial ischaemia. Extracellularly, [K^+^]_o_ is increased and pH is reduced. A rise in [K^+^]_o_ would initially cause membrane hyperpolarization, but as steady state is reached, there is a positive shift in the resting membrane potential as described by the Goldman field relationship [117]. It also increases the threshold potential to a smaller extent, thereby increasing myocardial excitability [117], [118]. This may explain why CV increases with mild increases in [K^+^]_o_ [119]. At higher concentrations, [K^+^]_o_ increases the proportion of inactivated sodium channels, which would reduce dV/dt_max_ and therefore the CV of the propagating AP [118], [120]. This is represented in Fig. 1 by an arrow originating from RMP and pointing to *I*_Na_. Thus, the effects of high [K^+^]_o_ on CV are biphasic, first increasing then decreasing the speed of conduction [119]. Experimental observations using microelectrode recordings indeed demonstrate reduced dV/dt_max_ during combined hyperkalaemia, acidosis and hypoxia in guinea pig ventricular muscle [49]. Extracellular acidification also reduces CV [121]. Intracellularly, increases in cyclic AMP and Ca^2 +^ have been described. Beta adrenergic stimulation increases cAMP and activation of protein kinase A [122], [123]. PKA in turn phosphorylates serine residues at 525 and 528 positions within the linker region between domains I and II [124], causing trafficking of sodium channels to the plasma membrane [125] and increase in *I*_Na_. However, there may also be a PKA-independent mechanism that reduces *I*_Na_, likely through a G-protein coupled signaling pathway. In contrast, calcium binds to the conserved C2 domain of protein kinase C [126], thereby activating it. PKC in turn phosphorylates the serine residue at 1505 position, located at the inactivation gate between domains III and IV, thereby reducing *I*_Na_ [127]. Together, all of the above changes produce decreases in both CV and action potential duration (APD), promoting arrhythmogenesis through a reduction in excitation wavelength given by the product of CV and effective refractory period (ERP, approximated by APD).

## 4 Gap junctions

Gap junctions were first described as hexagonal arrays of protein molecules in the plasma membrane of Mauthner cell synapses of goldfish [128]. They form high-resistance pathways for intercellular coupling, allowing passive, electrotonic spread of ions and also passage of larger molecules such as amino acids and nucleotides [129]. The resistance of gap junctions therefore contributes to r_i_ and determines CV [130], [131]. Each gap junction is made of two connexons, which are hexameric proteins of the connexin (Cx) subunit. In cardiac tissue, Cx 30.2, 40, 43 and 45 have been described [132]. Of these, Cx43 is expressed throughout the atria and ventricles [133], whereas Cx40 is expressed only in the atria and His–Purkinje system [134], [135]. Cx43 and Cx40 have unitary conductances of about 100 pS [136] and 160 pS [137], respectively. There are two major gating mechanisms, the conventional membrane voltage-dependent gating and also transjunctional voltage-dependent gating [138]. Its function is also regulated by phosphorylation [139], [140], [141], Ca^2 +^_i_ [142], [143], [144], [145], pH_i_ [146], [147] and the surrounding lipid environment [148], [149], [150], [151]. Gap junctions are also affected by exogenous substances such as lipophilic agents that uncouple them [152], [153], [154] or peptides enhancing their ionic conductances [155], [156].

Gap junctions represent the electrical coupling pathway between cardiomyocytes, whereas anchoring complexes termed fascia adherens junctions and desmosomes mediate mechanical coupling between them. Together these structures form the highly organized intercalated disk [158]. There is increasing evidence that sodium channels co-localize with gap junctions, and form the connexome, which includes a “protein-interacting network” of desmosomes, gap junctions and sodium channels [159]. What is becoming clear is that altered expression of one component influences the expression or localization of others. Thus, experiments using super-resolution fluorescence localization microscopy demonstrated that truncation of the C-terminus of Cx43 in adult murine cardiomyocytes resulted in reduced *I*_Na_ at the end of the cells [160]. Conversely, in heterozygous Scn5a-knockout mice with 50% reduction in sodium channel expression, disarrangement of gap junctions was observed [161].

### 4.1 Abnormalities in gap junction structure and function

Inherited mutations in GJA1, the gene that encodes for Cx43, have been associated with an autosomal dominant condition called oculodentodigital dysplasia [162], which predisposes to VT and sudden cardiac death. The effects of Cx43 loss on conduction and arrhythmogenesis have been extensively studied in mouse models [163], [164], [165], [166], [167], [168], [169], [170], [171], [172], [173], but not without considerable disagreement, as summarized previously [169]. Cardiac-restricted inactivation of Cx43 followed by crossing with Cre recombinase produced mice with mosacism, in which Cx43 was reduced by 86 to 95% compared to wild-type [163]. Their hearts were structurally normal by echocardiography but showed increased incidence of sudden death. In other studies, heterozygous Cx43^+/−^ mice showed 45 to 50% decrease in Cx43 expression. In these systems, CV was either unaltered [165], [166], [167], [169], [171], [172] or decreased by 23 to 44% [164], [168], [170]. Together, these studies suggest different parameters, such as intracellular calcium levels, interstitial volume [28], perinexal width and perfusate composition and osmolarity [169], influence the net behaviour, conduction, even in mice with identical genotype. In addition to conduction slowing, increased conduction dispersion is also a common abnormality following Cx43 downregulation [171], [174], [175]. This can refer to phase difference in conduction latencies of neighbouring regions [171], difference in CV across the myocardial wall [174] and dispersion represented by coefficient of dispersion, which uses standard deviation of the mean CV [175]. Finally, a mutation in plaktoglobin, a protein that links adjacent myocytes and anchors sarcomeres, has been observed in the recessive form of arrhythmogenic right ventricular dysplasia (ARVD), called Naxos disease, and has been associated with reduced gap junction expression in the myocardium [176].

Acquired abnormalities in gap junctions occur in several pathological conditions, such as ischaemia and infarction [177]. In the failing ventricle, tyrosine phosphorylation of Cx43 by c-Src tyrosine kinase is increased [178], reducing gap junction conductance. Additionally, heterogeneous distribution of Cx43 leads to increased dispersion of conduction and therefore re-entrant arrhythmogenesis [175]. The importance of Cx43 is supported by findings of its deletion following genetic modification, in which CV slowing and increased anisotropic conduction were observed [171]. In acute ischaemia, the following abnormalities have been detected. Increased Ca^2 +^_i_ has been associated with reduced conductance as well as uncoupling of gap junctions. PKC-mediated phosphorylation, a calcium-dependent process, of the serine residue at the 368 position, has been linked to reduced conductance [136], [141]. Dephosphorylation of gap junctions was also described [179], resulting in electrical uncoupling [180] and gap junction lateralization [181], [182]. In summary, the consequence of these changes in gap junction structure or function, whether in congenital or acquired disorders, would be to reduce CV and increase heterogeneity of conduction, hence predisposing to re-entrant excitation.

## 5 Therapeutic strategies

Understanding the mechanisms of conduction abnormalities allows effective pharmacological therapy to be developed for the treatment of ventricular arrhythmias. Several studies have focused on the arrhythmic effects of gap junction downregulation [169] or uncoupling [183]. However, protective effects of reducing infarct size as well as preventing arrhythmogenesis produced by ischaemia have also been shown for gap junction uncouplers [184], [185]. As demonstrated theoretically, in non-uniform tissue, mild loss of gap junction function paradoxically increases CV and improves the safety margin of conduction [16]. This can remove unidirectional blocks and therefore protect against arrhythmogenesis [32], [186], [187]. Indeed, experiments using cell strands connected to rectangular cell monolayers showed that partial gap junction uncoupling converted unidirectional conduction block to bidirectional conduction [188]. The therapeutic effects of gap junction uncouplers therefore warrant further investigation.

For heart failure, fibrosis is a significant pro-arrhythmic factor. In hypertensive rats, fibrosis can be prevented by the ACE inhibitor enalapril [189], the angiotensin II receptor blocker losartan [190] as well as the calcium channel blocker amlodipine [191]. In mouse hearts, the aldosterone receptor antagonist eplerenone and losartan both reduce fibrosis and arrhythmic tendency [192]. Fibrosis resulting from fibroblast activation is mediated by many protein growth factors, such as transforming growth factor beta [193]. This can be inhibited by the antifibrotic hormone relaxin [194]. To prevent enzymatic degradation, adenoviruses expressing relaxin can be injected into the body, and the relaxin can be detected in plasma [195]. This treatment has demonstrated efficacy in mice with fibrotic cardiomyopathy [195]. It may also be effective in individuals with Lenègre disease, Brugada syndrome and ARVD, in which myocardial fibrosis is present [97], [98], [99], [176], [196].

## 6 Conclusion

This article reviewed the physiological mechanisms by which alterations in structure and function of gap junctions and sodium channels lead to conduction abnormalities and in turn, to ventricular arrhythmogenesis. It is clear that AP conduction is not a simple phenomenon solely determined by biophysical parameters of ion channel conductances, and resistive and capacitive properties of myocytes. It is also influenced by myocardial cell size, ion channel localization, cellular architecture and source–sink relationships. For example, in heart failure, the extensive electrophysiological and structural remodelling are key factors in arrhythmogenesis. For instance, up- and downregulation of ion channels alter transmembrane currents, and fibrosis affects axial resistance and membrane capacitance. Conduction is a fascinating aspect of cardiac electrophysiology and there is much to be learnt. A systems approach is needed for the development of effective therapeutic agents that can prevent or abolish arrhythmias. Without a full appreciation of the pharmacological effects on the above factors, these agents may be at best, ineffective but at worst, will themselves induce malignant ventricular arrhythmias.

## References

[1] Adabag A.S., Luepker R.V., Roger V.L., Gersh B.J. (2010). Sudden cardiac death: epidemiology and risk factors. Nat. Rev. Cardiol..

[2] Zheng Z.J., Croft J.B., Giles W.H., Mensah G.A. (2001). Sudden cardiac death in the United States, 1989 to 1998. Circulation.

[3] Zipes D.P., Wellens H.J. (1998). Sudden cardiac death. Circulation.

[4] Go A.S., Hylek E.M., Phillips K.A., Chang Y., Henault L.E., Selby J.V. (2001). Prevalence of diagnosed atrial fibrillation in adults: national implications for rhythm management and stroke prevention: the AnTicoagulation and risk factors in atrial fibrillation (ATRIA) study. J. Am. Med. Assoc..

[5] Blackshear J.L., Kopecky S.L., Litin S.C., Safford R.E., Hammill S.C. (1996). Management of atrial fibrillation in adults: prevention of thromboembolism and symptomatic treatment. Mayo Clin. Proc..

[6] Rockson S.G., Albers G.W. (2004). Comparing the guidelines: anticoagulation therapy to optimize stroke prevention in patients with atrial fibrillation. J. Am. Coll. Cardiol..

[7] Marcus F.I. (2005). Depolarization/repolarization, electrocardiographic abnormalities, and arrhythmias in cardiac channelopathies. J. Electrocardiol..

[8] Kléber A.G., Rudy Y. (2004). Basic mechanisms of cardiac impulse propagation and associated arrhythmias. Physiol. Rev..

[9] Moe G.K. (1975). Evidence for reentry as a mechanism of cardiac arrhythmias. Rev. Physiol. Biochem. Pharmacol..

[10] Janse M.J., Wit A.L. (1989). Electrophysiological mechanisms of ventricular arrhythmias resulting from myocardial ischemia and infarction. Physiol. Rev..

[11] Mines G.R. (1913). On dynamic equilibrium in the heart. J. Physiol..

[12] Allessie M.A., Bonke F.I., Schopman F.J. (1973). Circus movement in rabbit atrial muscle as a mechanism of tachycardia. Circ. Res..

[13] Krishnan S.C., Antzelevitch C. (1993). Flecainide-induced arrhythmia in canine ventricular epicardium. Phase 2 reentry?. Circulation.

[14] Antzelevitch C., Jalife J., Moe G.K. (1980). Characteristics of reflection as a mechanism of reentrant arrhythmias and its relationship to parasystole. Circulation.

[15] Veeraraghavan R., Gourdie R.G., Poelzing S. (2014). Mechanisms of cardiac conduction: a history of revisions. Am. J. Physiol. Heart Circ. Physiol..

[16] Shaw R.M., Rudy Y. (1997). Ionic mechanisms of propagation in cardiac tissue. Roles of the sodium and L-type calcium currents during reduced excitability and decreased gap junction coupling. Circ. Res..

[18] Hodgkin A.L., Katz B. (1949). The effect of sodium ions on the electrical activity of giant axon of the squid. J. Physiol..

[19] Brismar T. (1977). Slow mechanism for sodium permeability inactivation in myelinated nerve fibre of *Xenopus laevis*. J. Physiol..

[20] Ruff R.L., Simoncini L., Stuhmer W. (1987). Comparison between slow sodium channel inactivation in rat slow- and fast-twitch muscle. J. Physiol..

[21] Furue T., Yakehiro M., Yamaoka K., Sumii K., Seyama I. (1998). Characteristics of two slow inactivation mechanisms and their influence on the sodium channel activity of frog ventricular myocytes. Pflugers Arch..

[22] Simoncini L., Stuhmer W. (1987). Slow sodium channel inactivation in rat fast-twitch muscle. J. Physiol..

[23] Ruff R.L., Simoncini L., Stuhmer W. (1988). Slow sodium channel inactivation in mammalian muscle: a possible role in regulating excitability. Muscle Nerve.

[24] Kootsey J.M., Glass L., Hunter P., McCulloch A.D. (1991). Electrical propagation in distributed cardiac tissue. Theory of Heart: Biomechanics, Biophysics, and Nonlinear Dynamics of Cardiac Function.

[25] Thomas S.P., Kucera J.P., Bircher-Lehmann L., Rudy Y., Saffitz J.E., Kleber A.G. (2003). Impulse propagation in synthetic strands of neonatal cardiac myocytes with genetically reduced levels of connexin43. Circ. Res..

[26] Rohr S., Kucera J.P., Kleber A.G. (1998). Slow conduction in cardiac tissue, I: effects of a reduction of excitability versus a reduction of electrical coupling on microconduction. Circ. Res..

[27] Weidmann S. (1952). The electrical constants of Purkinje fibres. J. Physiol..

[28] Veeraraghavan R., Salama M.E., Poelzing S. (2012). Interstitial volume modulates the conduction velocity-gap junction relationship. Am. J. Physiol. Heart Circ. Physiol..

[29] Spach M.S., Heidlage J.F., Barr R.C., Dolber P.C. (2004). Cell size and communication: role in structural and electrical development and remodeling of the heart. Heart Rhythm..

[30] Dhein S., Seidel T., Salameh A., Jozwiak J., Hagen A., Kostelka M. (2014). Remodeling of cardiac passive electrical properties and susceptibility to ventricular and atrial arrhythmias. Front. Physiol..

[31] Lin J., Keener J.P. (2013). Ephaptic coupling in cardiac myocytes. IEEE Trans. Biomed. Eng..

[32] Rohr S. (2004). Role of gap junctions in the propagation of the cardiac action potential. Cardiovasc. Res..

[33] Saffitz J.E., Kanter H.L., Green K.G., Tolley T.K., Beyer E.C. (1994). Tissue-specific determinants of anisotropic conduction velocity in canine atrial and ventricular myocardium. Circ. Res..

[34] Spach M.S. (2003). Transition from a continuous to discontinuous understanding of cardiac conduction. Circ. Res..

[35] Spach M.S., Miller W.T., Geselowitz D.B., Barr R.C., Kootsey J.M., Johnson E.A. (1981). The discontinuous nature of propagation in normal canine cardiac muscle. Evidence for recurrent discontinuities of intracellular resistance that affect the membrane currents. Circ. Res..

[36] Sano T., Takayama N., Shimamoto T. (1959). Directional difference of conduction velocity in the cardiac ventricular syncytium studied by microelectrodes. Circ. Res..

[37] Clerc L. (1976). Directional differences of impulse spread in trabecular muscle from mammalian heart. J. Physiol..

[38] Kumar N.M., Gilula N.B. (1996). The gap junction communication channel. Cell.

[39] Romero L., Trenor B., Ferrero J.M., Starmer C.F. (2013). Non-uniform dispersion of the source–sink relationship alters wavefront curvature. PLoS One.

[40] Huelsing D.J., Pollard A.E., Spitzer K.W. (2001). Transient outward current modulates discontinuous conduction in rabbit ventricular cell pairs. Cardiovasc. Res..

[41] Spitzer K.W., Pollard A.E., Yang L., Zaniboni M., Cordeiro J.M., Huelsing D.J. (2006). Cell-to-cell electrical interactions during early and late repolarization. J. Cardiovasc. Electrophysiol..

[42] Cohen S.A. (1996). Immunocytochemical localization of rH1 sodium channel in adult rat heart atria and ventricle. Presence in terminal intercalated disks. Circulation.

[43] Kucera J.P., Rohr S., Rudy Y. (2002). Localization of sodium channels in intercalated disks modulates cardiac conduction. Circ. Res..

[44] Veeraraghavan R., Lin J., Hoeker G.S., Keener J.P., Gourdie R.G., Poelzing S. (2015). Sodium channels in the Cx43 gap junction perinexus may constitute a cardiac ephapse: an experimental and modeling study. Pflugers Arch..

[45] Cerrone M., Delmar M. (2014). Desmosomes and the sodium channel complex: implications for arrhythmogenic cardiomyopathy and Brugada syndrome. Trends Cardiovasc. Med..

[46] Veeraraghavan R., Poelzing S., Gourdie R.G. (2014). Old cogs, new tricks: a scaffolding role for connexin43 and a junctional role for sodium channels?. FEBS Lett..

[47] Shaw R.M., Rudy Y. (1995). The vulnerable window for unidirectional block in cardiac tissue: characterization and dependence on membrane excitability and intercellular coupling. J. Cardiovasc. Electrophysiol..

[48] Coronel R. (1994). Heterogeneity in extracellular potassium concentration during early myocardial ischaemia and reperfusion: implications for arrhythmogenesis. Cardiovasc. Res..

[49] Kodama I., Wilde A., Janse M.J., Durrer D., Yamada K. (1984). Combined effects of hypoxia, hyperkalemia and acidosis on membrane action potential and excitability of guinea-pig ventricular muscle. J. Mol. Cell. Cardiol..

[50] Gellens M.E., George A.L.J., Chen L.Q., Chahine M., Horn R., Barchi R.L. (1992). Primary structure and functional expression of the human cardiac tetrodotoxin-insensitive voltage-dependent sodium channel. Proc. Natl. Acad. Sci. U. S. A..

[51] Stuhmer W., Conti F., Suzuki H., Wang X.D., Noda M., Yahagi N. (1989). Structural parts involved in activation and inactivation of the sodium channel. Nature.

[52] Kontis K.J., Rounaghi A., Goldin A.L. (1997). Sodium channel activation gating is affected by substitutions of voltage sensor positive charges in all four domains. J. Gen. Physiol..

[53] Horn R., Patlak J., Stevens C.F. (1981). Sodium channels need not open before they inactivate. Nature.

[54] West J.W., Patton D.E., Scheuer T., Wang Y., Goldin A.L., Catterall W.A. (1992). A cluster of hydrophobic amino acid residues required for fast Na(+)-channel inactivation. Proc. Natl. Acad. Sci. U. S. A..

[55] Kellenberger S., Scheuer T., Catterall W.A. (1996). Movement of the Na^+^ channel inactivation gate during inactivation. J. Biol. Chem..

[56] Kellenberger S., West J.W., Catterall W.A., Scheuer T. (1997). Molecular analysis of potential hinge residues in the inactivation gate of brain type IIA Na^+^ channels. J. Gen. Physiol..

[57] Kellenberger S., West J.W., Scheuer T., Catterall W.A. (1997). Molecular analysis of the putative inactivation particle in the inactivation gate of brain type IIA Na^+^ channels. J. Gen. Physiol..

[58] Smith M.R., Goldin A.L. (1997). Interaction between the sodium channel inactivation linker and domain III S4–S5. Biophys. J..

[59] Balser J.R., Nuss H.B., Chiamvimonvat N., Perez-Garcia M.T., Marban E., Tomaselli G.F. (1996). External pore residue mediates slow inactivation in mu 1 rat skeletal muscle sodium channels. J. Physiol..

[60] Vilin Y.Y., Makita N., George A.L., Ruben P.C. (1999). Structural determinants of slow inactivation in human cardiac and skeletal muscle sodium channels. Biophys. J..

[61] Gintant G.A., Datyner N.B., Cohen I.S. (1984). Slow inactivation of a tetrodotoxin-sensitive current in canine cardiac Purkinje fibers. Biophys. J..

[62] Carmeliet E. (1987). Slow inactivation of the sodium current in rabbit cardiac Purkinje fibres. Pflugers Arch..

[63] Patlak J.B., Ortiz M. (1985). Slow currents through single sodium channels of the adult rat heart. J. Gen. Physiol..

[64] Kiyosue T., Arita M. (1989). Late sodium current and its contribution to action potential configuration in guinea pig ventricular myocytes. Circ. Res..

[65] Wingo T.L., Shah V.N., Anderson M.E., Lybrand T.P., Chazin W.J., Balser J.R. (2004). An EF-hand in the sodium channel couples intracellular calcium to cardiac excitability. Nat. Struct. Mol. Biol..

[66] Mori M., Konno T., Ozawa T., Murata M., Imoto K., Nagayama K. (2000). Novel interaction of the voltage-dependent sodium channel (VDSC) with calmodulin: does VDSC acquire calmodulin-mediated Ca^2 +^-sensitivity?. Biochemistry.

[67] Van Petegem F., Lobo P.A., Ahern C.A. (2012). Seeing the forest through the trees: towards a unified view on physiological calcium regulation of voltage-gated sodium channels. Biophys. J..

[68] Chen-Izu Y., Shaw R.M., Pitt G.S., Yarov-Yarovoy V., Sack J.T., Abriel H. (2015). Na^+^ channel function, regulation, structure, trafficking and sequestration. J. Physiol..

[69] Wagner S., Dybkova N., Rasenack E.C., Jacobshagen C., Fabritz L., Kirchhof P. (2006). Ca^2 +^/calmodulin-dependent protein kinase II regulates cardiac Na^+^ channels. J. Clin. Investig..

[70] Dudel J., Peper K., Rudel R., Trautwein W. (1967). Effect of tetrodotoxin on membrane currents in mammalian cardiac fibres. Nature.

[71] Satin J., Kyle J.W., Chen M., Bell P., Cribbs L.L., Fozzard H.A. (1992). A mutant of TTX-resistant cardiac sodium channels with TTX-sensitive properties. Science.

[72] Sangameswaran L., Fish L.M., Koch B.D., Rabert D.K., Delgado S.G., Ilnicka M. (1997). A novel tetrodotoxin-sensitive, voltage-gated sodium channel expressed in rat and human dorsal root ganglia. J. Biol. Chem..

[73] Tzoumaka E., Tischler A.C., Sangameswaran L., Eglen R.M., Hunter J.C., Novakovic S.D. (2000). Differential distribution of the tetrodotoxin-sensitive rPN4/NaCh6/Scn8a sodium channel in the nervous system. J. Neurosci. Res..

[74] Klugbauer N., Lacinova L., Flockerzi V., Hofmann F. (1995). Structure and functional expression of a new member of the tetrodotoxin-sensitive voltage-activated sodium channel family from human neuroendocrine cells. EMBO J..

[75] Haufe V., Cordeiro J.M., Zimmer T., Wu Y.S., Schiccitano S., Benndorf K. (2005). Contribution of neuronal sodium channels to the cardiac fast sodium current I_Na_ is greater in dog heart Purkinje fibers than in ventricles. Cardiovasc. Res..

[76] Lei M., Jones S.A., Liu J., Lancaster M.K., Fung S.S., Dobrzynski H. (2004). Requirement of neuronal- and cardiac-type sodium channels for murine sinoatrial node pacemaking. J. Physiol..

[77] Maier S.K., Westenbroek R.E., Schenkman K.A., Feigl E.O., Scheuer T., Catterall W.A. (2002). An unexpected role for brain-type sodium channels in coupling of cell surface depolarization to contraction in the heart. Proc. Natl. Acad. Sci. U. S. A..

[78] Brette F., Orchard C.H. (2006). No apparent requirement for neuronal sodium channels in excitation-contraction coupling in rat ventricular myocytes. Circ. Res..

[79] Marban E. (2002). Cardiac channelopathies. Nature.

[80] Wang Q., Shen J., Li Z., Timothy K., Vincent G.M., Priori S.G. (1995). Cardiac sodium channel mutations in patients with long QT syndrome, an inherited cardiac arrhythmia. Hum. Mol. Genet..

[81] Bennett P.B., Yazawa K., Makita N., George A.L. (1995). Molecular mechanism for an inherited cardiac arrhythmia. Nature.

[82] Wang D.W., Yazawa K., George A.L., Bennett P.B. (1996). Characterization of human cardiac Na^+^ channel mutations in the congenital long QT syndrome. Proc. Natl. Acad. Sci. U. S. A..

[83] Dumaine R., Wang Q., Keating M.T., Hartmann H.A., Schwartz P.J., Brown A.M. (1996). Multiple mechanisms of Na^+^ channel–linked long-QT syndrome. Circ. Res..

[84] Carmeliet E. (1999). Cardiac ionic currents and acute ischemia: from channels to arrhythmias. Physiol. Rev..

[85] January C.T., Riddle J.M. (1989). Early afterdepolarizations: mechanism of induction and block. A role for L-type Ca^2 +^ current. Circ. Res..

[86] Szabo B., Sweidan R., Rajagopalan C.V., Lazzara R. (1994). Role of Na^+^:Ca^2 +^ exchange current in Cs(+)-induced early afterdepolarizations in Purkinje fibers. J. Cardiovasc. Electrophysiol..

[87] Liu M.B., de Lange E., Garfinkel A., Weiss J.N., Qu Z. (2015). Delayed afterdepolarizations generate both triggers and a vulnerable substrate promoting reentry in cardiac tissue. Heart Rhythm..

[88] Biet M., Morin N., Lessard-Beaudoin M., Graham R.K., Duss S., Gagne J. (2015). Prolongation of action potential duration and QT interval during epilepsy linked to increased contribution of neuronal sodium channels to cardiac late Na^+^ current: potential mechanism for sudden death in epilepsy. Circ Arrhythm Electrophysiol..

[89] George A.L. (2015). Misplaced brain sodium channels in heart kindle sudden death in epilepsy. Circ. Arrhythm. Electrophysiol..

[90] Chen Q., Kirsch G.E., Zhang D., Brugada R., Brugada J., Brugada P. (1998). Genetic basis and molecular mechanism for idiopathic ventricular fibrillation. Nature.

[91] Veldkamp M.W., Viswanathan P.C., Bezzina C., Baartscheer A., Wilde A.A., Balser J.R. (2000). Two distinct congenital arrhythmias evoked by a multidysfunctional Na(+) channel. Circ. Res..

[92] Litovsky S.H., Antzelevitch C. (1988). Transient outward current prominent in canine ventricular epicardium but not endocardium. Circ. Res..

[93] Wilde A.A., Postema P.G., Di Diego J.M., Viskin S., Morita H., Fish J.M. (2010). The pathophysiological mechanism underlying Brugada syndrome: depolarization versus repolarization. J. Mol. Cell. Cardiol..

[94] Antzelevitch C., Nof E. (2008). Brugada syndrome: recent advances and controversies. Curr. Cardiol. Rep..

[95] Postema P.G., van Dessel P.F., de Bakker J.M., Dekker L.R., Linnenbank A.C., Hoogendijk M.G. (2008). Slow and discontinuous conduction conspire in Brugada syndrome: a right ventricular mapping and stimulation study. Circ. Arrhythm. Electrophysiol..

[96] Benson D.W., Wang D.W., Dyment M., Knilans T.K., Fish F.A., Strieper M.J. (2003). Congenital sick sinus syndrome caused by recessive mutations in the cardiac sodium channel gene (SCN5A). J. Clin. Investig..

[97] Tan H.L., Bink-Boelkens M.T., Bezzina C.R., Viswanathan P.C., Beaufort-Krol G.C., van Tintelen P.J. (2001). A sodium-channel mutation causes isolated cardiac conduction disease. Nature.

[98] Schott J.J., Alshinawi C., Kyndt F., Probst V., Hoorntje T.M., Hulsbeek M. (1999). Cardiac conduction defects associate with mutations in SCN5A. Nat. Genet..

[99] Probst V., Kyndt F., Potet F., Trochu J.N., Mialet G., Demolombe S. (2003). Haploinsufficiency in combination with aging causes SCN5A-linked hereditary Lenegre disease. J. Am. Coll. Cardiol..

[100] Royer A., van Veen T.A., Le Bouter S., Marionneau C., Griol-Charhbili V., Leoni A.L. (2005). Mouse model of SCN5A-linked hereditary Lenegre's disease: age-related conduction slowing and myocardial fibrosis. Circulation.

[101] Remme C.A., Wilde A.A., Bezzina C.R. (2008). Cardiac sodium channel overlap syndromes: different faces of SCN5A mutations. Trends Cardiovasc. Med..

[102] Hothi S.S., Ara F., Timperley J. (2015). p.Y1449C SCN5A mutation associated with overlap disorder comprising conduction disease, Brugada syndrome, and atrial flutter. J. Cardiovasc. Electrophysiol..

[103] Liu M., Yang K.C., Dudley S.C. (2014). Cardiac sodium channel mutations: why so many phenotypes?. Nat. Rev. Cardiol..

[104] Gelband H., Bassett A.L. (1973). Depressed transmembrane potentials during experimentally induced ventricular failure in cats. Circ. Res..

[105] Ufret-Vincenty C.A., Baro D.J., Lederer W.J., Rockman H.A., Quinones L.E., Santana L.F. (2001). Role of sodium channel deglycosylation in the genesis of cardiac arrhythmias in heart failure. J. Biol. Chem..

[106] Valdivia C.R., Chu W.W., Pu J., Foell J.D., Haworth R.A., Wolff M.R. (2005). Increased late sodium current in myocytes from a canine heart failure model and from failing human heart. J. Mol. Cell. Cardiol..

[107] Pieske B., Houser S.R. (2003). [Na^+^]_i_ handling in the failing human heart. Cardiovasc. Res..

[108] Huang B., El-Sherif T., Gidh-Jain M., Qin D., El-Sherif N. (2001). Alterations of sodium channel kinetics and gene expression in the postinfarction remodeled myocardium. J. Cardiovasc. Electrophysiol..

[109] Moreno J.D., Clancy C.E. (2012). Pathophysiology of the cardiac late Na current and its potential as a drug target. J. Mol. Cell. Cardiol..

[110] Maltsev V.A., Reznikov V., Undrovinas N.A., Sabbah H.N., Undrovinas A. (2008). Modulation of late sodium current by Ca^2 +^, calmodulin, and CaMKII in normal and failing dog cardiomyocytes: similarities and differences. Am. J. Physiol. Heart Circ. Physiol..

[111] Lou Q., Janardhan A., Efimov I.R. (2012). Remodeling of calcium handling in human heart failure. Adv. Exp. Med. Biol..

[112] Ai X., Curran J.W., Shannon T.R., Bers D.M., Pogwizd S.M. (2005). Ca^2 +^/calmodulin-dependent protein kinase modulates cardiac ryanodine receptor phosphorylation and sarcoplasmic reticulum Ca^2 +^ leak in heart failure. Circ. Res..

[113] Gross G.J., Auchampach J.A. (1992). Role of ATP dependent potassium channels in myocardial ischaemia. Cardiovasc. Res..

[114] Hiraoka M. (1997). Pathophysiological functions of ATP-sensitive K^+^ channels in myocardial ischemia. Jpn. Heart J..

[115] Weiss J.N., Venkatesh N., Lamp S.T. (1992). ATP-sensitive K^+^ channels and cellular K^+^ loss in hypoxic and ischaemic mammalian ventricle. J. Physiol..

[116] Workman A.J., MacKenzie I., Northover B.J. (2000). Do KATP channels open as a prominent and early feature during ischaemia in the Langendorff-perfused rat heart?. Basic Res. Cardiol..

[117] Fisch C. (1973). Relation of electrolyte disturbances to cardiac arrhythmias. Circulation.

[118] Kishida H., Surawicz B., Fu L.T. (1979). Effects of K^+^ and K^+^-induced polarization on (dV/dt)max, threshold potential, and membrane input resistance in guinea pig and cat ventricular myocardium. Circ. Res..

[119] Buchanan J.W., Saito T., Gettes L.S. (1985). The effects of antiarrhythmic drugs, stimulation frequency, and potassium-induced resting membrane potential changes on conduction velocity and dV/dtmax in guinea pig myocardium. Circ. Res..

[120] Dominguez G., Fozzard H.A. (1970). Influence of extracellular K^+^ concentration on cable properties and excitability of sheep cardiac Purkinje fibers. Circ. Res..

[121] Veenstra R.D., Wang H.Z., Beyer E.C., Brink P.R. (1994). Selective dye and ionic permeability of gap junction channels formed by connexin45. Circ. Res..

[122] Bers D.M. (2002). Cardiac excitation-contraction coupling. Nature.

[123] Keely S.L. (1977). Activation of cAMP-dependent protein kinase without a corresponding increase in phosphorylase activity. Res. Commun. Chem. Pathol. Pharmacol..

[124] Murphy B.J., Rogers J., Perdichizzi A.P., Colvin A.A., Catterall W.A. (1996). cAMP-dependent phosphorylation of two sites in the alpha subunit of the cardiac sodium channel. J. Biol. Chem..

[125] Hallaq H., Yang Z., Viswanathan P.C., Fukuda K., Shen W., Wang D.W. (2006). Quantitation of protein kinase A-mediated trafficking of cardiac sodium channels in living cells. Cardiovasc. Res..

[126] Luo J.H., Weinstein I.B. (1993). Calcium-dependent activation of protein kinase C. The role of the C2 domain in divalent cation selectivity. J. Biol. Chem..

[127] Qu Y., Rogers J.C., Tanada T.N., Catterall W.A., Scheuer T. (1996). Phosphorylation of S1505 in the cardiac Na^+^ channel inactivation gate is required for modulation by protein kinase C. J. Gen. Physiol..

[128] Robertson J.D. (1963). The occurrence of a subunit pattern in the unit membranes of club endings in Mauthner cell synapses in goldfish brains. J. Cell Biol..

[129] Spray D.C., Burt J.M. (1990). Structure-activity relationships of the cardiac gap junction channel. Am. J. Physiol..

[130] Dhillon P.S., Gray R., Kojodjojo P., Jabr R., Chowdhury R., Fry C.H. (2013). Relationship between gap-junctional conductance and conduction velocity in mammalian myocardium. Circ. Arrhythm. Electrophysiol..

[131] Peters N.S. (2006). Gap junctions: clarifying the complexities of connexins and conduction. Circ. Res..

[132] Davis L.M., Kanter H.L., Beyer E.C., Saffitz J.E. (1994). Distinct gap junction protein phenotypes in cardiac tissues with disparate conduction properties. J. Am. Coll. Cardiol..

[133] Beyer E.C., Paul D.L., Goodenough D.A. (1987). Connexin43: a protein from rat heart homologous to a gap junction protein from liver. J. Cell Biol..

[134] Gourdie R.G., Green C.R., Severs N.J., Anderson R.H., Thompson R.P. (1993). Evidence for a distinct gap-junctional phenotype in ventricular conduction tissues of the developing and mature avian heart. Circ. Res..

[135] Gourdie R.G., Severs N.J., Green C.R., Rothery S., Germroth P., Thompson R.P. (1993). The spatial distribution and relative abundance of gap-junctional connexin40 and connexin43 correlate to functional properties of components of the cardiac atrioventricular conduction system. J. Cell Sci..

[136] Moreno A.P., Saez J.C., Fishman G.I., Spray D.C. (1994). Human connexin43 gap junction channels. Regulation of unitary conductances by phosphorylation. Circ. Res..

[137] Beblo D.A., Wang H.Z., Beyer E.C., Westphale E.M., Veenstra R.D. (1995). Unique conductance, gating, and selective permeability properties of gap junction channels formed by connexin40. Circ. Res..

[138] Bukauskas F.F., Verselis V.K. (2004). Gap junction channel gating. Biochim. Biophys. Acta.

[139] Musil L.S., Goodenough D.A. (1991). Biochemical analysis of connexin43 intracellular transport, phosphorylation, and assembly into gap junctional plaques. J. Cell Biol..

[140] Saez J.C., Nairn A.C., Czernik A.J., Fishman G.I., Spray D.C., Hertzberg E.L. (1997). Phosphorylation of connexin43 and the regulation of neonatal rat cardiac myocyte gap junctions. J. Mol. Cell. Cardiol..

[141] Kwak B.R., Hermans M.M., De Jonge H.R., Lohmann S.M., Jongsma H.J., Chanson M. (1995). Differential regulation of distinct types of gap junction channels by similar phosphorylating conditions. Mol. Biol. Cell.

[142] De Mello W.C. (1975). Effect of intracellular injection of calcium and strontium on cell communication in heart. J. Physiol..

[143] Dahl G., Isenberg G. (1980). Decoupling of heart muscle cells: correlation with increased cytoplasmic calcium activity and with changes of nexus ultrastructure. J. Membr. Biol..

[144] Burt J.M. (1987). Block of intercellular communication: interaction of intracellular H^+^ and Ca^2 +^. Am. J. Physiol..

[145] Maurer P., Weingart R. (1987). Cell pairs isolated from adult guinea pig and rat hearts: effects of [Ca^2 +^]i on nexal membrane resistance. Pflugers Arch..

[146] Hermans M.M., Kortekaas P., Jongsma H.J., Rook M.B. (1995). pH sensitivity of the cardiac gap junction proteins, connexin 45 and 43. Pflugers Arch..

[147] Morley G.E., Taffet S.M., Delmar M. (1996). Intramolecular interactions mediate pH regulation of connexin43 channels. Biophys. J..

[148] Meyer R., Malewicz B., Baumann W.J., Johnson R.G. (1990). Increased gap junction assembly between cultured cells upon cholesterol supplementation. J. Cell Sci..

[149] Meyer R.A., Lampe P.D., Malewicz B., Baumann W.J., Johnson R.G. (1991). Enhanced gap junction formation with LDL and apolipoprotein B. Exp. Cell Res..

[150] Massey K.D., Minnich B.N., Burt J.M. (1992). Arachidonic acid and lipoxygenase metabolites uncouple neonatal rat cardiac myocyte pairs. Am. J. Physiol..

[151] Schubert A.L., Schubert W., Spray D.C., Lisanti M.P. (2002). Connexin family members target to lipid raft domains and interact with caveolin-1. Biochemistry.

[152] Bernardini G., Peracchia C., Peracchia L.L. (1984). Reversible effects of heptanol on gap junction structure and cell-to-cell electrical coupling. Eur. J. Cell Biol..

[153] Deleze J., Herve J.C. (1983). Effect of several uncouplers of cell-to-cell communication on gap junction morphology in mammalian heart. J. Membr. Biol..

[154] Bastiaanse E.M., Jongsma H.J., van der Laarse A., Takens-Kwak B.R. (1993). Heptanol-induced decrease in cardiac gap junctional conductance is mediated by a decrease in the fluidity of membranous cholesterol-rich domains. J. Membr. Biol..

[155] Shiroshita-Takeshita A., Sakabe M., Haugan K., Hennan J.K., Nattel S. (2007). Model-dependent effects of the gap junction conduction-enhancing antiarrhythmic peptide rotigaptide (ZP123) on experimental atrial fibrillation in dogs. Circulation.

[156] Guerra J.M., Everett T.H., Lee K.W., Wilson E., Olgin J.E. (2006). Effects of the gap junction modifier rotigaptide (ZP123) on atrial conduction and vulnerability to atrial fibrillation. Circulation.

[158] Stroemlund L.W., Jensen C.F., Qvortrup K., Delmar M., Nielsen M.S. (2015). Gap junctions — guards of excitability. Biochem. Soc. Trans..

[159] Agullo-Pascual E., Cerrone M., Delmar M. (2014). Arrhythmogenic cardiomyopathy and Brugada syndrome: diseases of the connexome. FEBS Lett..

[160] Agullo-Pascual E., Lin X., Leo-Macias A., Zhang M., Liang F.X., Li Z. (2014). Super-resolution imaging reveals that loss of the C-terminus of connexin43 limits microtubule plus-end capture and NaV1.5 localization at the intercalated disc. Cardiovasc. Res..

[161] van Veen T.A., Stein M., Royer A., Le Quang K., Charpentier F., Colledge W.H. (2005). Impaired impulse propagation in Scn5a-knockout mice: combined contribution of excitability, connexin expression, and tissue architecture in relation to aging. Circulation.

[162] Kalcheva N., Qu J., Sandeep N., Garcia L., Zhang J., Wang Z. (2007). Gap junction remodeling and cardiac arrhythmogenesis in a murine model of oculodentodigital dysplasia. Proc. Natl. Acad. Sci. U. S. A..

[163] Gutstein D.E., Morley G.E., Tamaddon H., Vaidya D., Schneider M.D., Chen J. (2001). Conduction slowing and sudden arrhythmic death in mice with cardiac-restricted inactivation of connexin43. Circ. Res..

[164] Guerrero P.A., Schuessler R.B., Davis L.M., Beyer E.C., Johnson C.M., Yamada K.A. (1997). Slow ventricular conduction in mice heterozygous for a connexin43 null mutation. J. Clin. Investig..

[165] Stein M., van Veen T.A., Remme C.A., Boulaksil M., Noorman M., van Stuijvenberg L. (2009). Combined reduction of intercellular coupling and membrane excitability differentially affects transverse and longitudinal cardiac conduction. Cardiovasc. Res..

[166] Stein M., van Veen T.A., Hauer R.N., de Bakker J.M., van Rijen H.V. (2011). A 50% reduction of excitability but not of intercellular coupling affects conduction velocity restitution and activation delay in the mouse heart. PLoS One.

[167] Morley G.E., Vaidya D., Samie F.H., Lo C., Delmar M., Jalife J. (1999). Characterization of conduction in the ventricles of normal and heterozygous Cx43 knockout mice using optical mapping. J. Cardiovasc. Electrophysiol..

[168] Eloff B.C., Lerner D.L., Yamada K.A., Schuessler R.B., Saffitz J.E., Rosenbaum D.S. (2001). High resolution optical mapping reveals conduction slowing in connexin43 deficient mice. Cardiovasc. Res..

[169] George S.A., Sciuto K.J., Lin J., Salama M.E., Keener J.P., Gourdie R.G. (2015). Extracellular sodium and potassium levels modulate cardiac conduction in mice heterozygous null for the connexin43 gene. Pflugers Arch..

[170] Thomas S.A., Schuessler R.B., Berul C.I., Beardslee M.A., Beyer E.C., Mendelsohn M.E. (1998). Disparate effects of deficient expression of connexin43 on atrial and ventricular conduction: evidence for chamber-specific molecular determinants of conduction. Circulation.

[171] van Rijen H.V., Eckardt D., Degen J., Theis M., Ott T., Willecke K. (2004). Slow conduction and enhanced anisotropy increase the propensity for ventricular tachyarrhythmias in adult mice with induced deletion of connexin43. Circulation.

[172] Vaidya D., Tamaddon H.S., Lo C.W., Taffet S.M., Delmar M., Morley G.E. (2001). Null mutation of connexin43 causes slow propagation of ventricular activation in the late stages of mouse embryonic development. Circ. Res..

[173] Beauchamp P., Choby C., Desplantez T., de Peyer K., Green K., Yamada K.A. (2004). Electrical propagation in synthetic ventricular myocyte strands from germline connexin43 knockout mice. Circ. Res..

[174] Wiegerinck R.F., van Veen T.A., Belterman C.N., Schumacher C.A., Noorman M., de Bakker J.M. (2008). Transmural dispersion of refractoriness and conduction velocity is associated with heterogeneously reduced connexin43 in a rabbit model of heart failure. Heart Rhythm..

[175] Boulaksil M., Winckels S.K., Engelen M.A., Stein M., van Veen T.A., Jansen J.A. (2010). Heterogeneous connexin43 distribution in heart failure is associated with dispersed conduction and enhanced susceptibility to ventricular arrhythmias. Eur. J. Heart Fail..

[176] Kaplan S.R., Gard J.J., Protonotarios N., Tsatsopoulou A., Spiliopoulou C., Anastasakis A. (2004). Remodeling of myocyte gap junctions in arrhythmogenic right ventricular cardiomyopathy due to a deletion in plakoglobin (Naxos disease). Heart Rhythm..

[177] Wit A.L., Peters N.S. (2012). The role of gap junctions in the arrhythmias of ischemia and infarction. Heart Rhythm..

[178] Toyofuku T., Yabuki M., Otsu K., Kuzuya T., Tada M., Hori M. (1999). Functional role of c-Src in gap junctions of the cardiomyopathic heart. Circ. Res..

[179] Huang X.D., Sandusky G.E., Zipes D.P. (1999). Heterogeneous loss of connexin43 protein in ischemic dog hearts. J. Cardiovasc. Electrophysiol..

[180] Beardslee M.A., Lerner D.L., Tadros P.N., Laing J.G., Beyer E.C., Yamada K.A. (2000). Dephosphorylation and intracellular redistribution of ventricular connexin43 during electrical uncoupling induced by ischemia. Circ. Res..

[181] Smith J.H., Green C.R., Peters N.S., Rothery S., Severs N.J. (1991). Altered patterns of gap junction distribution in ischemic heart disease. An immunohistochemical study of human myocardium using laser scanning confocal microscopy. Am. J. Pathol..

[182] Lampe P.D., TenBroek E.M., Burt J.M., Kurata W.E., Johnson R.G., Lau A.F. (2000). Phosphorylation of connexin43 on serine368 by protein kinase C regulates gap junctional communication. J. Cell Biol..

[183] Tse G., Hothi S.S., Grace A.A., Huang C.L. (2012). Ventricular arrhythmogenesis following slowed conduction in heptanol-treated, Langendorff-perfused mouse hearts. J. Physiol. Sci..

[184] Li G., Whittaker P., Yao M., Kloner R.A., Przyklenk K. (2002). The gap junction uncoupler heptanol abrogates infarct size reduction with preconditioning in mouse hearts. Cardiovasc. Pathol..

[185] Chen B.P., Mao H.J., Fan F.Y., Bruce I.C., Xia Q. (2005). Delayed uncoupling contributes to the protective effect of heptanol against ischaemia in the rat isolated heart. Clin. Exp. Pharmacol. Physiol..

[186] Fast V.G., Kleber A.G. (1995). Block of impulse propagation at an abrupt tissue expansion: evaluation of the critical strand diameter in 2- and 3-dimensional computer models. Cardiovasc. Res..

[187] Leon L.J., Roberge F.A. (1991). Directional characteristics of action potential propagation in cardiac muscle. A model study. Circ. Res..

[188] Rohr S., Kucera J.P., Fast V.G., Kleber A.G. (1997). Paradoxical improvement of impulse conduction in cardiac tissue by partial cellular uncoupling. Science.

[189] Pahor M., Bernabei R., Sgadari A., Gambassi G., Lo Giudice P., Pacifici L. (1991). Enalapril prevents cardiac fibrosis and arrhythmias in hypertensive rats. Hypertension.

[190] Gay-Jordi G., Guash E., Benito B., Brugada J., Nattel S., Mont L. (2013). Losartan prevents heart fibrosis induced by long-term intensive exercise in an animal model. PLoS One.

[191] Sevilla M.A., Voces F., Carron R., Guerrero E.I., Ardanaz N., San Roman L. (2004). Amlodipine decreases fibrosis and cardiac hypertrophy in spontaneously hypertensive rats: persistent effects after withdrawal. Life Sci..

[192] Stein M., Boulaksil M., Jansen J.A., Herold E., Noorman M., Joles J.A. (2010). Reduction of fibrosis-related arrhythmias by chronic renin–angiotensin–aldosterone system inhibitors in an aged mouse model. Am. J. Physiol. Heart Circ. Physiol..

[193] Leask A. (2010). Potential therapeutic targets for cardiac fibrosis: TGFbeta, angiotensin, endothelin, CCN2, and PDGF, partners in fibroblast activation. Circ. Res..

[194] Samuel C.S., Unemori E.N., Mookerjee I., Bathgate R.A., Layfield S.L., Mak J. (2004). Relaxin modulates cardiac fibroblast proliferation, differentiation, and collagen production and reverses cardiac fibrosis in vivo. Endocrinology.

[195] Bathgate R.A., Lekgabe E.D., McGuane J.T., Su Y., Pham T., Ferraro T. (2008). Adenovirus-mediated delivery of relaxin reverses cardiac fibrosis. Mol. Cell. Endocrinol..

[196] Coronel R., Casini S., Koopmann T.T., Wilms-Schopman F.J., Verkerk A.O., de Groot J.R. (2005). Right ventricular fibrosis and conduction delay in a patient with clinical signs of Brugada syndrome: a combined electrophysiological, genetic, histopathologic, and computational study. Circulation.

